# Probing the internal structure of baryons

**DOI:** 10.1093/nsr/nwab187

**Published:** 2021-10-14

**Authors:** Guangshun Huang, Rinaldo Baldini Ferroli, BESIII Collaboration

**Affiliations:** Department of Modern Physics, University of Science and Technology of China, Hefei 230026, China; State Key Laboratory of Particle Detection and Electronics, Hefei 230026, China; INFN Laboratori Nazionali di Frascati, I-00044 Frascati, Italy; Institute of High Energy Physics, Chinese Academy of Sciences, Beijing 100049, China

**Keywords:** baryon structure, form factor, threshold effect, abnormal production

## Abstract

Electromagnetic form factors are fundamental observables that describe the electric and magnetic structure of hadrons and provide keys to understand the strong interaction. At the Beijing Spectrometer (BESIII), form factors have been measured for different baryons in the time-like region for the first time or with the best precision. The results are presented with examples focused on but not limited to the proton/neutron, the Λ, with a strange quark, and the Λ_*c*_, with a charm quark.

## Teaser

The internal electric and magnetic structure has been studied for a number of baryons by means of electromagnetic form factor measurements at the BESIII experiment.

## INTRODUCTION

Baryons and mesons are both hadrons, i.e. bound systems of quarks in a naive quark model [1] or, more accurately, also gluons in modern theory. Baryons are half-integer spin fermions, comprised, in a first approximation, of three quarks held together by the strong interactions. Protons (*p*) and neutrons (*n*), collectively known as nucleons (*N*), are the lightest baryons, and are the major components of the observable matter of the Universe. A nucleon has three valence light quarks (*u* or *d*); if one or more of its *u* or *d* quarks are replaced by heavier quarks (*s*, *c*, *b* or *t*), it becomes a hyperon. The most known baryons are the spin 1/2 SU(3) octet, including the isospin doublet *p*/*n*, singly stranged isospin singlet Λ, singly stranged isospin triplet Σ^−^/Σ^0^/Σ^+^ and the doubly stranged isospin doublet Ξ^−^/Ξ^0^ [2]. The lightest charmed baryon is the $\Lambda _c^+$ [2]. Hadrons are not point-like particles, and their internal electric and magnetic structure is characterized by their electromagnetic form factors (FFs).

**Figure 1.**
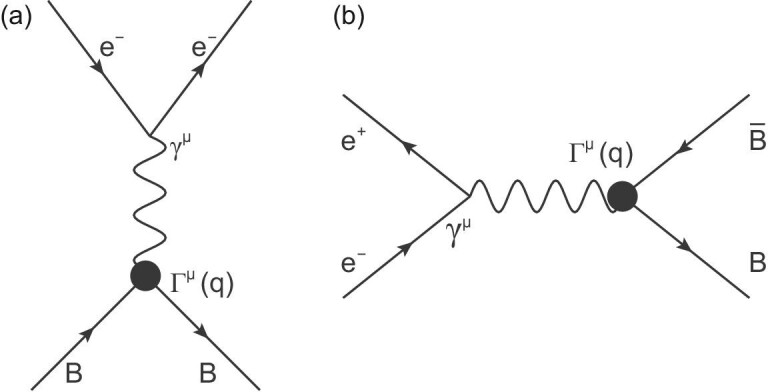
Lowest-order Feynman diagrams for elastic electron-hadron scattering *e*^−^*B* → *e*^−^*B* (a), and for the annihilation process $e^+e^- \rightarrow B \bar{B}$ (b).

The particles are so tiny (of the order of 10^−15^ m, or a femtometre) that they cannot be observed directly by the human eye (ability of 10^−4^ m, or 0.1 mm), an optical microscope (resolution of 10^−7^ m, or 0.1 μm) or even an electric microscope (resolution of 10^−10^ m, or 0.1 nm, the size of an atom). Instead, their properties are studied through collisions. When two particles traverse each other, they interact by exchanging force carriers called bosons that transfer some energy and momentum (i.e. four momentum) from one to the other. For electron-nucleon scattering, the electron is a probe that spies the secrets hidden inside the nucleon, and in this case the four-momentum transfer squared has a negative value (*q*^2^ < 0), and is categorized as a space-like process. When a particle and an anti-particle meet, for example in the case of an electron and a positron, they can annihilate—i.e. disappear into a virtual photon—and then produce an fermion-antifermion pair that eventually materializes as a system of hadrons, of which a baryon-antibaryon pair is one possibility. In this case the four-momentum transfer squared has a positive value (*q*^2^ > 0), and is classified as a time-like process. The Feynman diagrams for these two processes are shown in Fig. 1(a) and (b), respectively. For the latter, the form factors of the participating baryon can be deduced from the behavior of the outgoing baryon-antibaryon pair, which is the subject of the study covered in this paper.

Hadronic production data from electron-positron annihilations at low energies (around the giga-electron-volt order) are important to the understanding of the structure of hadrons and the strong interactions of their constituent quarks. Moreover, since hyperons are not stable, they can be studied only in the time-like domain. The Beijing Spectrometer (BESIII) [3] at the Beijng Electron Positron Collider (BEPCII) [4] operates in the center-of-mass energy range from 2.0 and 4.6 GeV, which is a transition region between perturbative and non-perturbative quantum chromodynamics (QCD). Using the initial state radiation (ISR) technique, BESIII can also access energies below 2.0 GeV. The *e*^+^*e*^−^ collision data that are used for QCD studies at BESIII include an integrated luminosity of 12 pb^−1^ at four energies (2.23, 2.4, 2.8 and 3.4 GeV) in the continuum taken in 2012, about 800 pb^−1^ at 104 energies between 3.85 and 4.6 GeV taken in the 2013–14 run, and about 650 pb^−1^ at 22 energies from 2.0 to 3.08 GeV taken in 2015. These are the so-called scan data, with moderate luminosity at each energy point; nonetheless, for these energies, they are the largest data samples in the world. There are also much larger samples for charm physics or XYZ particle search, some as large as a few fb^−1^ at a single energy, which are suitable for ISR-type analyses. With these huge data samples, BESIII is uniquely well suited to make baryon form factor measurements with unprecedentedly high precision.

## BARYON MYSTERIES

The standard wisdom is that baryons are bound states of three quarks, but this description is incomplete. For example, though nucleons are the basic building blocks of observable matter in the Universe, not all of their basic properties such as their size, spin, magnetic moment and mass are fully understood, even after 100 years of study [5,6].

The charge radius of a proton measured by muonic Lamb shift once differed from that determined by electron-proton scattering or electronic Lamb shift by as much as five standard deviations [7], but recent measurements from electron scattering [8] and hydrogen spectroscopy [9] eliminated the discrepancies, and this so-called proton-radius puzzle has been essentially solved [10,11].

The proton spin has also been in a crisis in the era of the constituent quark model. The European Muon Collaboration (EMC) experiment found that the baryon spin is not only due to the spins of the valence quark [12]. It has been commonly assumed that the proton’s spin of 1/2 was formed by two quarks with parallel spins and a third quark with opposite spin. In the EMC experiment, a quark of a polarized proton target was struck by a polarized muon beam, and the quark’s instantaneous spin was measured. It was expected that the spin of two of the three quarks would cancel out and the spin of the third quark would be polarized in the direction of the proton’s spin. Thus, the sum of the quarks’ spin was expected to be equal to the proton’s spin. Surprisingly, it was found that the number of quarks with spin in the proton’s spin direction was almost the same as the number of quarks whose spin was in the opposite direction. Similar results have been obtained in many experiments afterwards, demonstrating clearly that both generalized parton distributions and transverse momentum distributions are important in the nucleon spin structure [13]. Our modern understanding is that the nucleon spin comes not only from quarks but also from gluons, and various contributions can be calculated using, e.g. Ji’s sum rule [14]. The abnormal magnetic moment of a proton (much larger than that for a Dirac point-like particle) is generally considered an indication of a more complicated internal structure than simply three spin-1/2 quarks in a relative *S* wave.

Moreover, the mass of a proton cannot be explained by the Higgs mechanism, since the sum of the quarks masses inside a proton is too small, which means that there are considerable contributions to its mass from the strong interactions among quarks and gluons. Nowadays, these contributions can be calculated precisely in the lattice QCD, so the proton mass is largely understood [15,16].

## BARYON FORM FACTOR MEASUREMENTS AT BESIII

The differential cross section of electron-positron annihilation to a baryon-antibaryon pair can be written as a function of the center-of-mass (c.m.) energy squared *s* as [17]

(1) \begin{eqnarray*} \frac{d\sigma _{B\bar{B}} (s)}{d\Omega } &=&\frac{\alpha ^{2}\beta C}{4s}\bigg [|G_{M}(s)|^2(1+\cos ^{2}\theta )\nonumber \\ &&+\frac{4m_{B}^{2}}{s}|G_{E}(s)|^2\sin ^{2}\theta \bigg ], \end{eqnarray*}

where θ is the polar angle of the baryon in the *e*^+^*e*^−^ c.m. frame and $\beta = \sqrt{1-4m_{B}^2/s}$ is the speed of the baryon. The Gamov-Sommerfeld factor *C* [18–20] describes the Coulomb enhancement effect: for a charged baryon pair, *C* = *y*/(1 − e^−*y*^) with $y = \pi \alpha \sqrt{1-\beta ^{2}} / \beta$ accounts for the electromagnetic interaction between the outgoing baryons; while for a neutral baryon pair, *C* = 1. The form factors, *G*_*E*_ and *G*_*M*_, essentially describe the electric and magnetic distributions inside the baryon and basically provide a measure of its boundary or size. These are functions of the four-momentum transfer *s* = *q*^2^, so should more accurately be written as *G*_*E*_(*q*^2^) and *G*_*M*_(*q*^2^). In the time-like domain, the form factors are complex with nonzero imaginary parts, and the translation into the internal structure is not straightforward, contrary to the case in the space-like region. It is noteworthy that final-state interactions become prevailing close to threshold and should thus be properly dealt with. By definition, the electric and magnetic form factors should be equal at the baryon-antibaryon pair’s mass threshold where only *s*-wave production contributes [21], i.e. $G_E(4m_B^2)=G_M(4m_B^2)$, but generally they are not. In analyses of data with limited statistics it is often assumed that they are equal and the two form factors are replaced by an effective form factor, *G*_eff_ = *G*_*E*_ = *G*_*M*_.

In principle, the Coulomb interaction between the outgoing charged baryon pair *B*^+^*B*^−^ should play an important role, in particular, by producing an abrupt jump in the cross section at threshold, since the phase space factor β is canceled by a 1/β factor in the Coulomb correction (however, there is no full consensus on that), which is a non-perturbative correction to the Born approximation to account for the Coulomb interaction between the outgoing charged baryons. In fact, the cross section for $e^+e^- \rightarrow p\bar{p}$ at threshold has been measured to be very close to the point-like value, which is consistent with the prediction, but then it is followed by a flat behavior, which is unexpected. While, for a neutral-baryon pair $B^0\bar{B}^0$, the cross section at threshold should be zero according to equation (1). The minimum c.m. energy for BESIII data is 2.0 GeV, which is about 122 MeV above the nucleon-antinucleon threshold, so no solid conclusion can be drawn for the proton-pair and neutron-pair cases, but BESIII can test these effects for charged baryons by seeing if there is a step with a value close to the point-like one for $\Lambda _c^+\bar{\Lambda }_c^-$ production, and for neutral baryons by seeing if the cross section is vanishing at the $\Lambda \bar{\Lambda }$ at threshold. Present BESIII results seem to indicate that at both the $\Lambda _c^+\bar{\Lambda }_c^-$ and $\Lambda \bar{\Lambda }$ thresholds there is a step that is close to the point-like value for charged particles, although maybe not exactly the same.

### Proton

**Figure 2.**
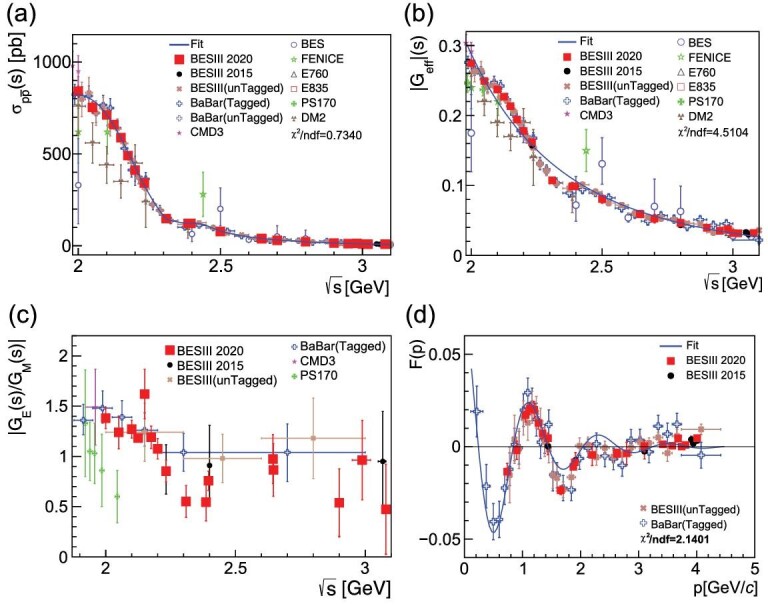
(a) The cross sections for $e^+e^- \rightarrow p\bar{p}$. (b) The effective proton time-like form factor. The blue curve is the result of an attempt to fit the measurements with a smooth dipole-like function. (c) The ratio *R* = |*G*_*E*_/*G*_*M*_|. (d) Effective form factor residual *F*(*p*) after subtracting the one calculated by QCD theory (the blue curve shown in (b)), as a function of the relative motion *p* of the final proton and antiproton. Plots are from [37].

Space-like proton form factors have been measured with very high precision in many experiments [22,23]. In the time-like region, there have been a few measurements of *G*_eff_ by DM2 [24,25], E760 [26], PS170 [27], FENICE [28], E835 [29,30], BaBar [31,32] and CMD-3 [33,34], but these have relatively poor precision and mutual agreement. For the |*G*_*E*_/*G*_*M*_| ratio, the measurements were rare and there is a long-time tension between PS170 and BaBar. The BESII experiment also measured the proton effective form factor, but with poor statistical precision [35]. BESIII continued this effort using the 2012 and 2015 scan data, and produced the most accurate |*G*_*E*_/*G*_*M*_| ratio measurements at 16 c.m. energies between 2.0 and 3.08 GeV [36,37] that favor BaBar over PS170 and helped clarify the puzzle. BESIII also performed the measurements using the ISR technique [38,39], with results that are consistent with those of BaBar. The BESIII measurements are shown in panel (a) of Fig. 2 for a $p\bar{p}$ production cross section in the range 2.0–3.08 GeV, in panel (b) for the effective proton time-like form factor, in panel (c) for the form factor ratio *R* = |*G*_*E*_/*G*_*M*_| and in panel (d) for the effective form factor residual, together with results from other experiments. The best precision in the time-like region was reported by BESIII, and the electric form factor was extracted for the first time. The unprecedented 3.5% uncertainty that was achieved at 2.125 GeV by BESIII is close to that of the best measurements in the space-like region, which have been at per cent levels for a long time. The CMD-3 experiment measured the production cross section of the proton pair and observed an abrupt rise at the nucleon-antinucleon threshold [34], as expected for point-like charged particles according to equation (1). BESIII did not extend down to the threshold energy, but the results around 2 GeV agree with CMD-3. This information improves our understanding of the proton inner structure from a different dimension and helps us to test theoretical models that depend on non-perturbative QCD, e.g. charge distribution within the proton can be deduced [40,41]. The near threshold behavior of the electromagnetic form factor of a hadron is mostly determined by the interaction of the hadron and antihadron in the final state, and therefore measurements of the form factor properties can also serve as a fruitful source of information about hadron-antihadron interaction [42].

Interestingly there are oscillations in the effective proton form factor, first seen by BaBar and later confirmed by BESIII [38]. These oscillations were subsequently studied with more precise data by BESIII [37]. Bianconi and Tomasi-Gustafsson [43] speculated that possible origins of this curious behavior are rescattering processes at relative distances of 0.7–1.5 fm between the centers of the forming hadrons, leading to a large fraction of inelastic processes in *p*-$\bar{p}$ interactions, and a large imaginary component to the rescattering processes.

### Neutron

**Figure 3.**
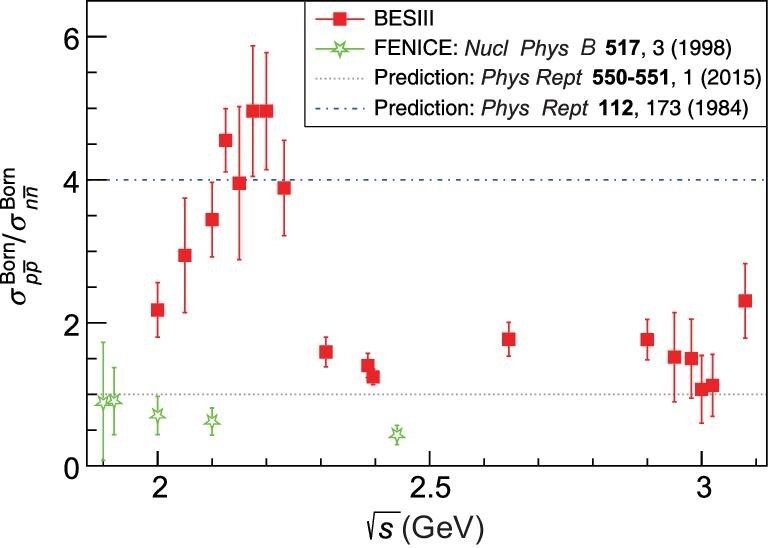
Ratio of the Born cross section of $e^+e^- \rightarrow p\bar{p}$ to that of $e^+e^- \rightarrow n\bar{n}$.

Prior to the BESIII experiment, there was a long-standing puzzle related to the differences between the neutron and proton production rates. QCD-motivated models predict that the cross section for the proton should be 4 times larger than for the neutron [44], or they should be same [45]. In contrast, the FENICE experiment found that the neutron cross section was twice as large as the proton’s, albeit with statistics that were very limited, only 74 $n\bar{n}$ events in total for five energy bins [28]. More recent measurements in the vicinity of the nucleon-antinucleon threshold are from the SND experiment [46,47]. The cross sections of $e^+e^- \rightarrow n\bar{n}$ and the neutron form factors between 2 and 3.08 GeV have been measured by BESIII with a good deal more data, over 2000 $n\bar{n}$ events at 18 energies [48]. Because the final-state neutron and anti-neutron are both neutral, with no tracks recorded in the drift chamber, the event selection is a challenge. The information in the calorimeter and the time-of-flight counters has to be used to identify the signal; as such, the selection efficiency is much lower and the number of observed neutron events is significantly less than that for protons. Neutron measurements from SND [46,47] and BESIII [48] overlap and roughly agree at 2 GeV, where a cross-section  behavior that is close to the $e^+e^- \rightarrow p\bar{p}$ case is observed, in particular, a flat behavior above threshold up to 2 GeV, as seen by CMD-3 [34]; however, this challenges the expected behavior from equation (1). For energies above 2 GeV, the BESIII measurements of the ratio of the proton-to-neutron cross sections is more compatible with the QCD-motivated model predictions: as shown in Fig. 3, the cross section for $e^+e^- \rightarrow p\bar{p}$ is larger than for $e^+e^- \rightarrow n\bar{n}$ in general.

From BESIII measurements of the angular distributions for $e^+e^- \rightarrow N\bar{N}$ events, the *S*-wave and *D*-wave contributions are disentangled for the first time, which is currently under further investigation in the collaboration. Moreover, from comparisons of the $e^+e^- \rightarrow n\bar{n}$ and $e^+e^- \rightarrow p\bar{p}$ cross sections, the isoscalar and isovector components of $e^+e^- \rightarrow N\bar{N}$ can, in principle, be separated [49]. One of the components dominates and is nearly constant up to 2 GeV, similar to $e^+e^- \rightarrow p\bar{p}$, but at present it is difficult to identify whether this component is the isoscalar (very likely the largest) or the isovector one. With more data in the future, this identification could be achieved by BESIII.

### The **Λ** hyperon

**Figure 4.**
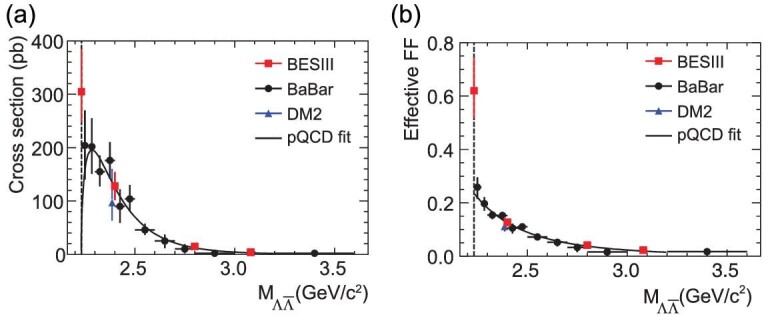
(a) Measurements of the $e^+e^{-} \rightarrow \Lambda \bar{\Lambda }$ cross section. (b) The Λ effective form factor. Plots are from [51].

The Λ, which is the lightest hyperon that contains an *s* quark, is more difficult to study than the nucleon because of its smaller production cross section. It was measured previously in the DM2 [25] and BaBar [50] experiments, but the results were not conclusive. BESIII has studied the channel $e^+e^{-} \rightarrow \Lambda \bar{\Lambda }$ [51] with an analysis that used a 40.5 pb^−1^ data sample that was collected at four different energy scan points during 2011 and 2012. The lowest energy point is 2.2324 GeV, only 1 MeV above the $\Lambda \bar{\Lambda }$ threshold. These data made it possible to measure the Born cross section very near threshold. To use the data as efficiently as possible, both events where Λ and $\bar{\Lambda }$ decayed to the charged mode ($\mathrm{Br}(\Lambda \rightarrow p\pi ^-) = 64\%$) and events where the $\bar{\Lambda }$ decayed to the neutral mode ($\mathrm{Br}(\bar{\Lambda } \rightarrow \bar{n} \pi ^0) = 36\%$) were selected. In the first case, the identification relied on finding two mono-energetic charged pions with evidence for a $\bar{p}$ annihilation in the material of the beam pipe or the inner wall of the tracking chamber. In the second case, the $\bar{n}$ annihilation was identified with a multi-variate analysis of variables provided by the electromagnetic calorimeter. Additionally, a mono-energetic π^0^ was reconstructed to fully identify this decay channel. For the higher energy points, only the charged decay modes of Λ and $\bar{\Lambda }$ were reconstructed by identifying all the charged tracks and using the event kinematics. The resulting measurements [51] of the Born cross section are shown in Fig. 4(a) together with previous measurements [25,50]. The Born cross section near threshold is found to be $312 \pm 51(\rm stat.) ^{+72}_{-45}$(sys.) pb. This result confirms BaBar’s measurement [50], but with much higher momentum transfer squared accuracy. Since the Coulomb factor is equal to 1 for neutral baryon pairs, the cross section is expected to go to zero at threshold. Therefore, the observed threshold enhancement implies the existence of a complicated underlying physics scenario. The unexpected features of baryon pair production near threshold have driven a lot of theoretical studies, including scenarios that invoke bound states or unobserved meson resonances [42,52,53]. It was also interpreted as an attractive Coulomb interaction on the constituent quark level [54,55]. Another possible explanation is that the final-state interactions play an important role near the threshold [56–58]. The BESIII measurement improves previous results at low invariant masses at least by 10$\%$ and even more above 2.4 GeV/c. The Λ effective form factor extracted from the cross-section measurement is shown in Fig. 4(b).

**Figure 5.**
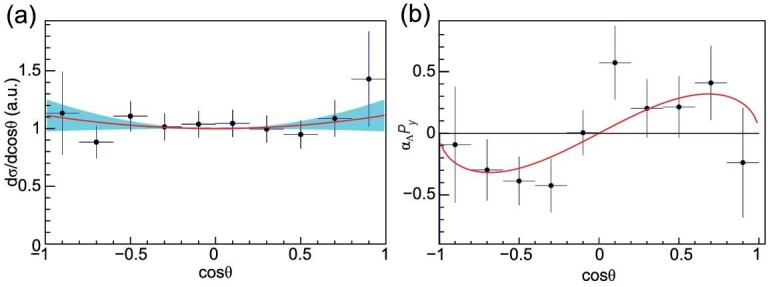
(a) The acceptance corrected Λ scattering angle distribution for $e^+e^{-} \rightarrow \Lambda \bar{\Lambda }$ at $M_{\Lambda \bar{\Lambda }} = 2.396$ GeV. (b) The product of the Λ decay parameter $\alpha _\Lambda$ and Λ polarization *P*_*y*_ as a function of the scattering angle. Plots are from [59].

According to the optical theorem, there is a nonzero relative phase between *G*_*E*_ and *G*_*M*_. At $M_{\Lambda \bar{\Lambda }} = 2.396$ GeV, where we have the largest $\Lambda \bar{\Lambda }$ sample of 555 events from 66.9 pb^−1^ data, a multi-dimensional analysis was used to make a full determination of the Λ electromagnetic form factors for the first time for any baryon; the relative phase difference is ΔΦ = 37° ± 12° ± 6° [59] with the input parameter $\alpha _\Lambda = 0.750\pm 0.010$ measured from *J*/ψ decays [60]. The improved determination of $\alpha _\Lambda$ also has profound implications for the baryon spectrum, since fits to such observables by theoretical models are a crucial element in determining the light baryon resonance spectrum, which provides a point of comparison for theoretical approaches [61]. The |*G*_*E*_/*G*_*M*_| ratio was determined to be $R = 0.96 \pm 0.14(\rm stat.) \pm 0.02(\rm sys.)$ and the effective form factor at $M_{\Lambda \bar{\Lambda }} = 2.396$ GeV was determined to be $|G_{\rm eff}| = 0.123 \pm 0.003 (\rm stat.) \pm 0.003 (\rm sys.)$. The Λ angular distribution and the polarization as a function of the scattering angle are shown in Fig. 5(a) and (b), respectively. This first complete measurement of the hyperon electromagnetic form factor is a milestone in the study of the hyperon structure, while the long-term goal is to describe charge and magnetization densities of the hyperons.

### The **Λ**_*c*_ charmed baryon

**Figure 6.**
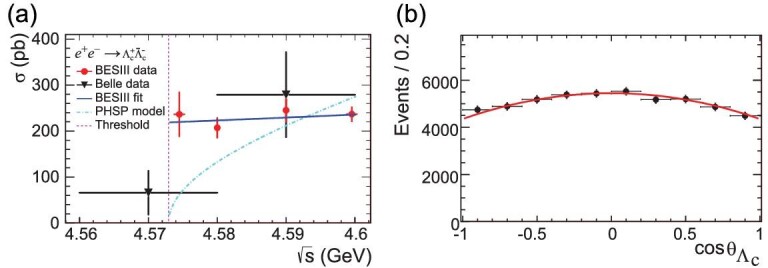
(a) The Born cross section of $e^+e^- \rightarrow \Lambda _c^+ \bar{\Lambda }_c^-$ obtained by BESIII and Belle. (b) The angular distribution and corresponding fit results in data at $\sqrt{s} = 4.5995$ GeV. Plots are from [63].

Experimental studies on charmed baryons have been rather sparse. The only previous study of the process $e^+e^- \rightarrow \Lambda _c^+ \bar{\Lambda }_c^-$ is from the Belle experiment, which measured the cross section using the ISR technique [62], and reported a lineshape that implied the existence of a likely resonance, called the Y(4660). Based on 631.3 pb^−1^ data collected in 2014 at the four energy points $\sqrt{s} =$ 4.5745, 4.5809, 4.5900 and 4.5995 GeV, BESIII measured the $\Lambda _c^+ \bar{\Lambda }_c^-$ cross section with unprecedented precision [63]. The lowest energy point is only 1.6 MeV above the $\Lambda _c^+ \bar{\Lambda }_c^-$ threshold. At each of the energy points, ten Cabibbo-favored hadronic decay modes, $\Lambda _c^+ \rightarrow p K^-\pi ^+$, $p K_S^0$, Λπ^+^, *pK*^−^π^+^π^0^, *pK*^0^π^0^, Λπ^+^π^0^, *pK*_*S*_π^+^π^−^, Λπ^+^π^+^π^−^, Σ^0^π^+^ and Σ^+^π^+^π^−^, as well as the corresponding charge-conjugate modes were studied. The total Born cross section is obtained from the weighted average of the 20 individual measurements, and the results are shown in Fig. 6(a). Similar to the case for $e^+e^- \rightarrow p \bar{p}$, an abrupt rise in the cross section just above threshold that is much steeper than phase-space expectations is discerned, which was not seen by Belle due to limitations in the ISR method. BESIII’s measured cross-section lineshape is different from Belle’s, disfavoring a resonance like Y(4660) in the $\Lambda _c^+ \bar{\Lambda }_c^-$ channel. The BESIII results have driven discussions in the theoretical literature [64].

High statistic data samples at $\sqrt{s} =$ 4.5745 and 4.5995 GeV enabled studies of the polar angular distribution of Λ_*c*_ in the *e*^+^*e*^−^ center-of-mass system. The shape function $f(\theta ) \propto (1 + \alpha _{\Lambda _c}\cos ^2\theta )$ is fitted to the combined data containing the yields of $\Lambda _c^+$ and $\bar{\Lambda }_c^-$ for all ten decay modes, as shown in Fig. 6(b). The ratio between the electric and magnetic form factors |*G*_*E*_/*G*_*M*_| can be extracted using $|G_E/G_M|^2(1-\beta ^2) = (1 - \alpha _{\Lambda _c})/ (1 + \alpha _{\Lambda _c})$. From these distributions, the ratios |*G*_*E*_/*G*_*M*_| of $\Lambda _c^+$ have been extracted for the first time: they are $1.14 \pm 0.14\,(\rm stat.) \pm 0.07\,(\rm sys.)$ and $1.23 \pm 0.05\,(\rm stat.) \pm 0.03\,(\rm sys.)$ at $\sqrt{s} =$ 4.5745 and 4.5995 GeV, respectively.

## BARYON CHALLENGES AT BESIII

**Figure 7.**
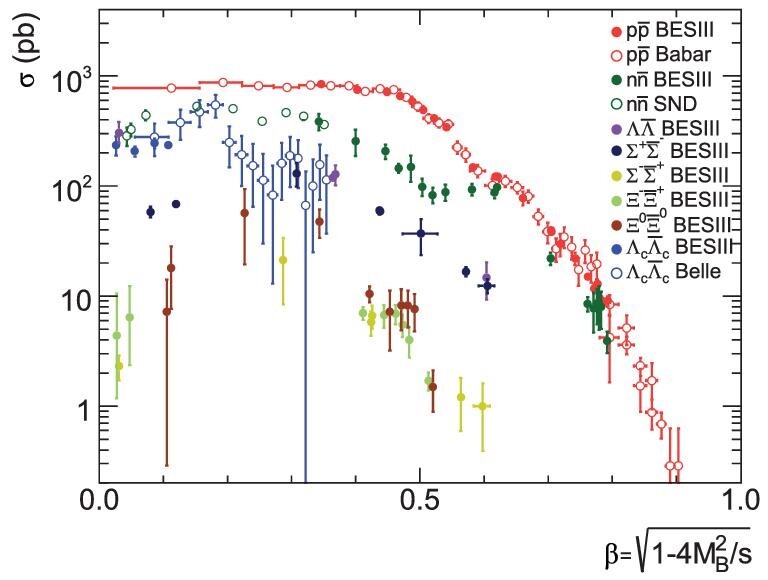
A compilation of cross sections, revealing similar patterns for all $B \bar{B}$ pairs measured so far: $p\bar{p}$ by BaBar [31,32] and BESIII [37], $n\bar{n}$ by SND [46,47] and BESIII [48], $\Lambda \bar{\Lambda }$ by BESIII [51], $\Sigma ^{+}\bar{\Sigma }^{-}$/$\Sigma ^{-}\bar{\Sigma }^{+}$ by BESIII [65], $\Xi ^{-}\bar{\Xi }^{+}$/$\Xi ^{0}\bar{\Xi }^{0}$ by BESIII [66,67], $\Lambda _c^+\bar{\Lambda }_c^-$ by Belle [62] and BESIII [63].

The energy thresholds for pair production of all of the ground-state spin-1/2 SU(3) octet and spin-3/2 decuplet are accessible to BESIII. Baryon form factor measurements are among the most important reasons why BESIII has collected an unprecedented amount of off-resonance data. From the analysis of existing data, it is expected that the ratio of the absolute values of the Λ electromagnetic form factors, |*G*_*E*_/*G*_*M*_|, can be measured at five energy points. The most interesting findings are the abrupt cross-section jumps at threshold followed by a nearly flat behavior that has been observed for $\Lambda \bar{\Lambda }$, $\Lambda _c^+\bar{\Lambda }_c^-$, $p\bar{p}$, $n\bar{n}$, etc. If the BEPCII energy could be lowered to the vicinity of nucleon-antinucleon threshold, BESIII will be able to confirm the $p\bar{p}$ and $n\bar{n}$ cases with much better precision. Figure 7 shows the cross-section lineshapes for a variety of baryon-antibaryon pairs, including those that were recently measured for singly stranged $\Sigma ^{+}\bar{\Sigma }^{-}$/$\Sigma ^{-}\bar{\Sigma }^{+}$ [65], doubly stranged $\Xi ^{-}\bar{\Xi }^{+}$ [66] and $\Xi ^{0}\bar{\Xi }^{0}$ [67]. They all seem to share the common feature of a plateau starting from the baryon pair production threshold, though for some channels, more statistics are ideally needed. The behaviors of $\Sigma ^{0}\bar{\Sigma }^{0}$ (the last member to be covered for the spin-1/2 SU(3) octet baryons) and other baryon pairs will be reported in the near future.

## SUMMARY AND PROSPECTS

The measurements of baryon form factors have been an important ongoing activity at BESIII. Form factors of the proton with the best precision were obtained in the time-like region, and the electric form factor of the proton was measured for the first time. Measurements of the neurtron time-like form factor with unprecedented precision have also been reported. The Λ and Λ_*c*_ were studied and in both cases abnormal cross-section enhancements were observed near the production thresholds. The form factors of the Λ_*c*_ were extracted for the first time.

In addition, Σ^+^/Σ^−^ [65], Ξ^−^ [66] and Ξ^0^ [67] form factor measurements were recently reported, and results for Σ^0^ will soon be released. BESIII also has a plan to explore the nucleon production threshold by taking data in the range 1.8–2.0 GeV for ∼100 pb^−1^ at 23 energy points [68], in order to study the anomalous threshold cross-section behavior in more detail. With numerous first measurements and interesting discoveries, these studies shed new light on the understanding of interactions and the fundamental structure of particles.

It will take a long time to ultimately unravel the fundamental structure of baryons. Further improvements in the form factor measurement of baryons will continue to be the focus of future powerful electron-ion colliders in America (EiC) [69] and China (EicC) [70], super electron-positron colliders in China [71] and Russia [72] for the space-like and time-like regions, respectively.
